# A Comparative Study on the Aerobic Biodegradation of the Biopolymer Blends of Poly(butylene succinate), Poly(butylene adipate terephthalate) and Poly(lactic acid)

**DOI:** 10.3390/polym14091894

**Published:** 2022-05-05

**Authors:** Nomvuyo Nomadolo, Omotola Esther Dada, Andri Swanepoel, Teboho Mokhena, Sudhakar Muniyasamy

**Affiliations:** 1 Centre for Nanostructures and Advanced Materials, Council for Scientific and Industrial Research (CSIR), Pretoria 0001, South Africa; nnomadolo@csir.co.za (N.N.); abswanepoel@csir.co.za (A.S.); 2 Department of Chemistry, Nelson Mandela University, Port Elizabeth 6000, South Africa; 3 Department of Biological Sciences, Elizade University, Ilara-Mokin 340271, Nigeria; omotola.dada@elizadeuniversity.edu.ng; 4 DSI-Nanotechnology Innovation Centre, Advanced Materials Division, Mintek, Randburg 2194, South Africa; mokhenateboho@gmail.com

**Keywords:** biopolymer, biopolymer blends, PLA, PBAT, PBS, biodegradability

## Abstract

The aim of the present work is to evaluate the rate and mechanisms of the aerobic biodegradation of biopolymer blends under controlled composting conditions using the CO_2_ evolution respirometric method. The biopolymer blends of poly (butylene adipate terephthalate) (PBAT) blended with poly (lactic acid) (PLA), and PBAT blended with poly (butylene succinate) (PBS) by melt extrusion, were tested to evaluate the amount of carbon mineralized under home and industrial composting conditions. The changes in the structural, chemical, thermal and morphological characteristics of the biopolymer blends before and after biodegradation were investigated by FT-IR, DSC, TGA, XRD and SEM. Both blends showed higher degradation rates under industrial composting conditions, when compared to home composting conditions. This was confirmed by FT-IR analysis showing an increase in the intensity of hydroxyl and carbonyl absorption bands. SEM revealed that there was microbial colony formation and disintegration on the surfaces of the biopolymer blends. The obtained results suggest that industrial composting conditions are the most suitable for an enhanced biodegradation of the biopolymer blends viz PBAT–PBS and PBAT–PLA.

## 1. Introduction

Globally, annual plastic production currently exceeds 335 million tons. Approximately 50–55% of these plastics are widely used for single-use and short-term purposes, with much of them being discarded indiscriminately or disposed of in landfills [1]. The extensive application of plastics has led to the rise of ‘white’ waste accumulation, which poses serious environmental problems due to the non-degradability in both terrestrial and aquatic environments [2,3]. Biodegradable and compostable polymers offer an alternative, because they can degrade in a defined period of time without having a negative environmental impact. Some biodegradable polymers of synthetic and natural origins, such as polylactic acid (PLA), polybutylene adipate terephthalate (PBAT), polyhydroxybutyrate (PHB), chitosan, polybutylene succinate (PBS) and poly(3-caprolactone) (PCL), are becoming more promising structural materials to replace certain single-use non-biodegradable plastic products [2,3]. Additionally, the use of biodegradable polymers supports resource conversion and reduces greenhouse emissions [4].

Many studies showed that industrial applications of biodegradable and compostable polymers present some drawbacks, due to their poor thermal and mechanical properties, which limits wider industrial sectors, such as packaging, agricultural and medical applications. However, these drawbacks can be addressed overcome by polymer modification techniques, either by polymer melt blending and other chemical synthesis routes to design and improve various thermal and mechanical properties, in order to be comparable to conventional plastics. In recent years, the design and development of innovative biodegradable and compostable products by polymer modification techniques received unprecedented interest from industrial and research communities as suitable replacements for conventional petroleum-based polymers by various sectors [5,6,7,8].

Poly (butylene adipate-co-terephthalate) (PBAT) has been reported to exhibit significant biodegradation rates, whether in the form of films or molded objects with competitive mechanical properties, in comparison to commodity plastics [5,6]. It is an ideal biodegradable polymer due to the excellent physical properties of the material, such as its high elongation at break, high flexibility, good thermal and chemical resistance, good tear resistance, as well as good hydrophilic and processing properties [7,8]. PBAT applications include the production of packaging materials, hygiene and biomedical products [8]. PBAT, however, exhibits low stiffness, which limits its wide application. Researchers made efforts to improve the mechanical performance, while maintaining the biodegradability and impact-resistance properties. Thus, the blending of PBAT with other biodegradable polymers, such as PLA and PBS, has been reported as a suitable solution to improve its stiffness [9,10].

Polylactic acid (PLA) is a polyester derived from renewable resources. It can degrade into carbon dioxide, water and microbial biomass under composting conditions [11,12]. In recent years, PLA-based materials have been widely used due to their unique properties, such as biodegradability and the possibility of modifications suitable for various applications [3]. However, the high cost of PLA, a high degree of brittleness and low levels of degradation in the natural environment significantly hamper its commercial application. Studies have shown that blending PBAT with PLA reduced the overall costs, while improving the overall biodegradation and mechanical properties of PLA [13].

Another biodegradable aliphatic polyester with high flexibility, excellent impact strength and easy processability is poly butylene succinate (PBS). PBS can degrade within several months in the environment [11,12,13]. The blending of these biodegradable thermoplastics reduces the overall costs, while also overcoming the impairments of the individual biopolymers. It also presents an opportunity to fine tune the degradation rate of the resulting material. In addition to the fact that there are numerous studies based on the mechanical properties and morphological changes of the polymer blends of PLA, PBAT and PBS, there is little information on the biodegradation behavior of these blends, especially the biodegradation mechanisms under home and industrial composting conditions [6,13,14,15,16,17,18].

In the literature, in the biodegradation studies of polymeric materials, one of the most commonly used techniques is the weight loss of the sample, however, the weight loss measurement of the sample is not directly related with true biodegradation, i.e., measuring the polymeric conversion into CO_2_. The weight loss of the polymeric materials is a primary degradation step chain scission of long-chain molecule into smaller oligomers and monomers influenced by various environmental abiotic (e.g., heat, sunlight, and humidity) and/or biotic actors (e.g., enzymatic). Among the available biodegradation test methods, the assessment of CO_2_ evolution from test samples is the most direct method, when compared to the other indirect tests, such as weight loss, surface deterioration and microbial colony formation. Monitoring the CO_2_ release from the action of polymeric materials by microorganisms is direct evidence of ultimate biodegradation (mineralization), in contrast to primary deterioration or fragmentation step. This CO_2_ biodegradation test method is globally accepted by established standards, such as ISO, ASTM and the European normative framework, for claiming environmental friendly polymeric materials. Therefore, the present study of CO_2_ biodegradation techniques was followed for studying the biodegradation of PLA/PBT/PBS composites under home and industrial composting conditions [18,19].

According to the American Society for Testing and Materials (ASTM), compostable plastic is ‘a plastic material that undergoes degradation by biological processes during composting to yield carbon dioxide, water, inorganic compounds, and biomass at a rate similar to well-known compostable materials without leaving behind any visually distinguishable or toxic residues’ [19]. It is worth mentioning that a compostable plastic is biodegradable, whereas a biodegradable plastic is not always compostable [20]. The landfilling of solid waste is the most common method of waste disposal throughout the world, and it remains the most economic form of disposal in many countries, especially in developing countries. The key role of solid-waste landfills is to receive and store municipal waste, of which a significant portion is plastic. Unfortunately, the rate of post-consumer plastic waste accumulation is far greater than the rate of natural biodegradation and, as a result, landfills are quickly filling up, leading to increased water, air and soil pollution. Moreover, with most landfills, particularly in developing countries, no measures exist to prevent gas and leachate emissions into the environment [21]. Therefore, the composting of biodegradable plastics aids in decreasing the need for landfill use by directing waste away from landfills [22,23] as well as contributing to efforts to reduce methane emissions. Improved soil quality as a result of nutrient enrichment provided by composting can also result in the expansion of agricultural activities [22].

The aim of this work is to investigate the aerobic biodegradation behavior of the PBAT–PLA and PBAT–PBS blends, under home and industrial composting conditions. Very few studies have been undertaken on the ultimate biodegradability of biobased polymers, such as PBS, PBAT and PLA. According to our knowledge, there are no significant studies that investigate the CO_2_ evolution of biopolymer blends, including the monitoring of the physical, chemical and thermal properties before and after biodegradation via a mechanistic approach. Therefore, the aim of this study is to evaluate the conversion of polymeric carbon into CO_2_ of the test samples under controlled composting conditions. Differential scanning calorimetry (DSC), Fourier Transform Infrared Spectroscopy (FT-IR), X-ray diffraction (XRD) and scanning electron microscopy (SEM) are also employed to monitor and quantitatively describe the degradation process.

## 2. Materials and Methods

### 2.1. Materials

The PBS (BioPBS FZ91 grade) used in this study was obtained from PTTMCC, Bangkok, Thailand. PBAT (Ecolfex C1200 grade) was obtained from BASF, Ludwigshafen, Germany and the PLA (PLA LX175 grade) was obtained from Total Corbion, Rayong, Thailand. For the biodegradation studies, 100% organic compost was obtained from a local nursery in Pretoria, South Africa.

### 2.2. The Preparation and Processing of the Biopolymer Blends

The biopolymer blends PBAT–PBS (30/70) and PBAT–PLA (80/20) were prepared by twin-screw melt extrusion. These ratios were found to be the optimal blending ratios that met the required melt flow index (MFI) and mechanical properties suitable for blown film applications. Before processing, all the biopolymer materials were pre-dried at 80 °C for 8 hrs. The PBAT–PBS (30/70) blend was processed at temperatures of 120–160 °C with a screw speed of 45 rpm. The PBAT–PLA (80/20) blend was processed between 140 °C and 180 °C, with a screw speed of 45 rpm. The extruded pellets were dried in an oven at 80 °C for 8 h. This was followed by the solution casting of the polymer blends dissolved in a chloroform solvent at an ambient temperature to obtain test sample films with an average thickness of 20–25 microns.

### 2.3. Biodegradation Testing

The method used to determine the biodegradability was based on the ASTM D 6400 and ASTM D5338 standard methods for home composting at 28 °C and industrial composting at 58 °C, respectively. The compost material was sieved to a diameter of less than 0.8 cm, and its physical and chemical properties were analyzed. The results are summarized in Table 1.

The biodegradability of the test materials was analyzed in biometer-respirometric flasks, according to a published method [19,20]. The sieved compost was mixed with finely ground perlite particles in a 1:1 dry weight ratio, which served to maintain proper humidity and aerobic conditions, as well as to provide noise elimination in the test. The polymer test materials were cut into 3 × 4 cm^2^ samples. The test samples were added to the compost mixture in a ratio of 1:6 (*w*/*w* sample to dry solids of compost). The mixture was placed on a bottom layer of 20 g of perlite, wetted with 15 g of water, followed with an upper layer of 20 g of perlite, and again wetted with 15 g of water. This perlite arrangement helps to maintain uniform humidity in the compost mixture, as well as eliminate noise in the test results.

The test blank (compost) and test samples (compost + test sample) were each carried out in three replicates. A 0.5 M potassium hydroxide (KOH) solution was placed on the upper layer of the compost mixture in the air-tight biometer flasks to trap the CO_2_ evolved from the test samples. The respirometer flasks with the mixtures were stored at 28 °C (room temperature), as well as in an oven at 58 °C. At intervals of 2–4 days, the KOH solution was withdrawn and analyzed via titration with a hydrochloric acid solution to determine the amount of evolved CO_2_. After titration, fresh 0.5 M KOH solution was added to the test flasks. Throughout the test conditions, the compost moisture content was maintained at a relative humidity of 50–55%. The degree of biodegradation was calculated from the amounts of CO_2_ produced by the added sample minus the amount of respiration CO_2_ generated by the blank.

The total CO_2_ emitted by each sample during the biodegradation studies was taken as representative of the total degradation due to biological factors. The total organic carbon of the polymer samples (C_t_) was determined by elemental analysis. Equation (1) was used to calculate the theoretical CO_2_ (CO_2_(t)) in the total dry weight of the plastic material.

(1) $$CO_{2\;}\left(t\right)=M_{t}\times C_{t}\times \frac{44}{12}\;$$

M_t_ is the total dry weight of plastic material added to the compost, and C_t_ is the relative weight of the total organic carbon in the dry plastic material. The degree of biodegradation for each test sample was calculated as a percentage of the overall theoretical CO_2_ (Equation (2)), where (CO_2_)s and (CO_2_)c are the amounts of CO_2_ produced in the sample and in the control (blank), respectively.

(2) $$Biodegradation\left(\%\right)=\frac{\left(CO_{2}\right)s-\left(CO_{2}\right)c}{\left(CO_{2}\right)t}\times 100\;$$

### 2.4. The Analytical Characterization

*Differential Scanning Colorimetry:* A DSCQ 2000 differential scanning calorimeter (Advanced Laboratory Solutions, Easton, PA, USA) was employed to determine the melting temperature (T_m_) and heat of fusion (ΔH_m_) during the degradation process. The samples were weighed and analyzed under a nitrogen atmosphere at a heating rate of 10 °C/min. The degree of crystallinity (X_c_) was calculated by Equation (3), with the enthalpy of 100% crystalline PBAT ($\Delta H_{m100})$, 100% crystalline PLA ($\Delta H_{m100})$ and 100% crystalline PBS ($\Delta H_{m100})$ taken as 114 J/g, 93.7 J/g and 110 J/g, respectively [24,25,26].

(3) $$X_{c}=\frac{\Delta H_{m}}{\Delta H_{m100}}\times 100$$

*Fourier Transform Infrared Spectroscopy (FT-IR):* FT-IR analyses were performed using a Spectrum 100 (Perkin Elmer, Waltham, MA, USA) at ambient temperature. The samples were analyzed in ATR spectra mode within a wavelength range of 4000–400 cm^−1^. These analyses were performed before degradation and after 30 and 60 days of degradation. The carbonyl index (I_CO_) of a sample was calculated from the ratio between the area underneath the carbonyl peak and the area below the -CH- peak, as in Equation (4) [5]:

(4) $$\%I_{CO}=\frac{A_{C-O}}{A_{C-H}}\;\times \;100\;$$

*Thermogravimetric Analysis:* A thermogravimetric analyzer (TGA), Pyris (PerkinElmer), was used to analyze the thermal degradation behavior of the test samples between 30 °C and 700 °C at a heating rate of 10 °C/min in a nitrogen atmosphere.

*X-Ray Diffraction (XRD):* The XRD measurements of the test samples before and after biodegradation were performed using a Rigaku Ultima IV X-ray diffractometer with Cu-Kα radiation (λ = 0.15406 nm).

*Scanning Electron Microscopy (SEM):* SEM (JEOL 7500) was used to determine the surface morphology of the test samples (coated with chromium) before and after biodegradation. The images were captured using an acceleration voltage of 3kV at a working distance of 8 mm.

## 3. Results and Discussion

### 3.1. Biodegradation Stimulated to Industrial Composting Conditions

**Figure 1:**
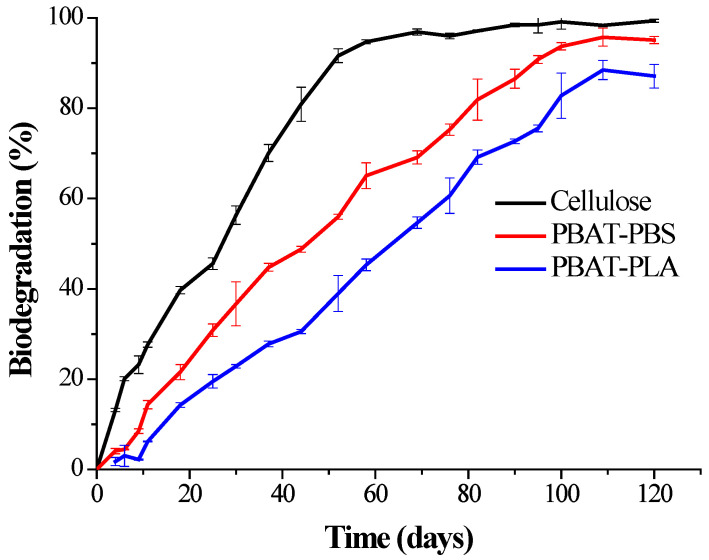
The biodegradation behavior of the PBAT–PLA and PBAT–PBS blends and cellulose (positive reference) under industrial composting conditions.

The biodegradation results for the PBAT–PLA and PBAT–PBS blends, in comparison to cellulose (positive reference) under industrial composting conditions, are shown in Figure 1. The biological activity during the compost biodegradation analysis was evaluated by measuring the CO_2_ emissions. The blank compost generated 90 mg of CO_2_ per gram of volatile solids in the first 10 days, which indicated that the compost medium had active microorganisms and could be utilized for biodegradation studies of polymeric materials. Moreover, the cellulose-positive reference material showed 70% biodegradation within 45 days, thus providing the sufficient authentication of the adopted test procedure and meeting the ASTM D6400 standard requirements [19].

The biodegradation of the PBAT–PBS blend showed a short lag phase for the first 8 days under composting conditions. This is typical for polymer degradation tests and could be attributed to the microbes in the compost requiring time to acclimatize to the new environment [10,23]. The lag phase was followed by a second phase, in which accelerated mineralization occurred between the 11th day and 80th days. The degree of biodegradation increased from ~18% to ~82%, respectively. The key drive behind this second accelerated phase was attributed to the fact that the material acted as an excellent carbon source for microbial growth, due to its low molecular weight, thus serving as substrate for enzymatic hydrolysis [24].

The biodegradation profile of the PBAT–PLA blend also showed a similar lag phase during the first 10 days, followed by a steady increase in the degree of biodegradation. The rate of degradation for the PBAT-PLA sample was lower than that of the PBAT-PBS sample. This lower rate of hydrolysis of the PBAT–PLA blend could be attributed to the extended degradation of the highly crystalline PLA component. The degree of biodegradation reached ~90% within 120 days under controlled composting conditions.

### 3.2. FT-IR Spectroscopy

**Figure 2:**
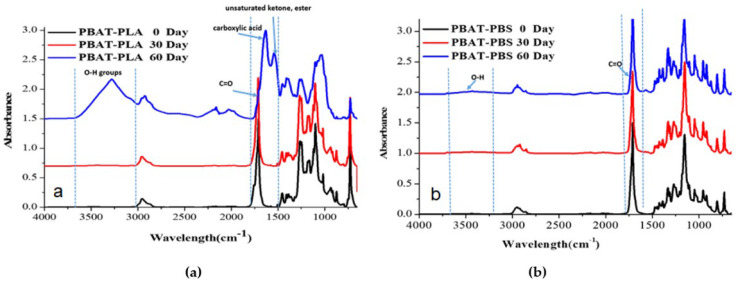
(**a**) The IR spectra of PBAT–PLA at 0 day, 30 days and 60 days after degradation, and (**b**) the IR spectra of PBAT–PBS at 0 day, 30 days and 60 days after degradation.

FT-IR spectra for the PBAT–PLA and PBAT–PBS blends before and after biodegradation at 30 days and 60 days are shown in Figure 2.

The PBAT–PLA blend showed an absorption band corresponding to the C–O stretching peak at wavenumber 1710 cm^−1^, which can be associated with the presence of PBAT in the blend (see Figure 2a) [25]. The absorption band at wavenumbers 1262 cm^−1^ and 1168 cm^−1^ corresponded to the symmetric vibration of C–O for PBAT and the symmetric vibrational peak of C–O–C for PLA, respectively. The absorption band at wavenumber 873 cm^−1^ corresponded to the O–CH–CH3 PLA ester group [5]. The two key functional groups that were studied were the hydroxyl (OH) and carbonyl (C–O) groups, to trace the degradation process. This is because biodegradation leads to a main chain scission at ester linkages from hydrolysis producing terminal alcohol and carboxylic acid groups. As hydrolysis continues, an increase in the OH groups should thus be observed in the FT-IR absorbance spectra, as the degradation progresses [26]. After 60 days of biodegradation, significant changes to the FT-IR spectrum of the PBAT–PLA blend were observed. The peak intensities for the characteristic absorption bands decreased, when compared to the neat sample. A shift of the characteristic peak for C–O from 1710 cm^−1^ to 1632 cm^−1^ occurred. Moreover, the biodegradation process led to the appearance of a new peak at 3280 cm^−1^ for the OH groups. As shown in Figure 2b, the PBAT–PBS IR spectra displayed similar characteristic peaks to those of the PBAT–PLA spectra, except that the carbonyl peaks shifted slightly from 1710 cm^−1^ to 1712 cm^−1^, which could be due to the presence of PBS [27]. The degradation of the PBAT–PBS blend is reflected by the reduction in the characteristic peak intensities between the 0 day sample and the 60 days sample. This could be attributed to the reduction in the molecular weight and changes in the chemical structure through abiotic hydrolysis [27,28]. The ester linkages of PBS and PBAT are more sensitive to elevated temperature and moisture [28], and therefore undergo hydrolytic degradation through the cleavage of the ester linkages on the polymer backbone. Additionally, the hydrolysis reaction may occur in the form of a depolymerization process and a random chain scission mechanism [27].

Table 2 shows the carbonyl index (Ico) calculated for the PBAT–PLA and PBAT–PBS blends using the ratio of the CH peak at 1452 cm^−1^ to the CO peak between 1710 cm^−1^–1712 cm^−1^. The I_CO_ values for the PBAT–PLA blend increased from 4.41 to 9.88. These results suggest that the degradation of the PBAT–PLA blend started with hydrolytic chain scission [29,30,31]. The chain scission led to the formation of short polymer chains, which, in turn, resulted in a large number of carboxylic acid end groups. These end groups further facilitated the degradation process of the entire blend [30,32,33]. The I_CO_ value of the PBAT–PBS blend, however, decreased from 4.95 to 3.32, indicating that a different mechanism was involved. The decrease in the I_CO_ values indicated that there was an increase in the assimilation of the carbonyl peaks by microbes. This could be attributed to three degradation mechanisms simultaneously taking place, namely, hydrolytic chain scission, main chain scission and β-C–H hydrogen transfer [30,32].

### 3.3. TGA Analyses

**Figure 3:**
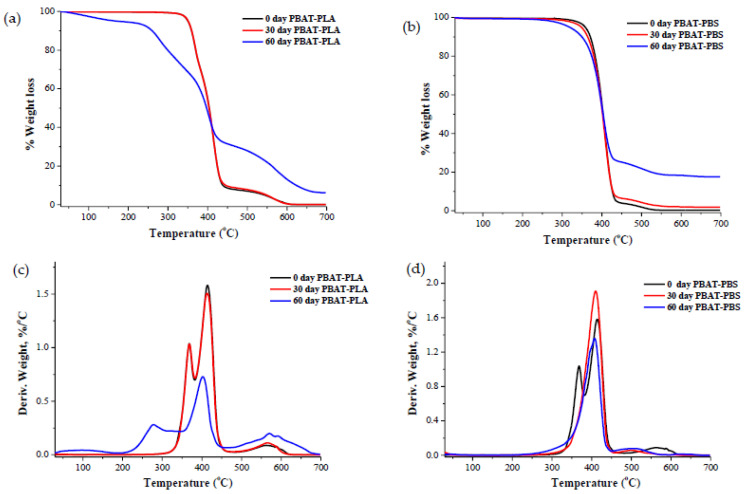
(**a**) TGA curves of the PBAT–PLA samples before, during and after degradation; (**b**) TGA curves of the PBAT–PBS samples before, during and after degradation; (**c**) the DTG curves of the PBAT–PLA samples before, during and after degradation; and (**d**) the DTG curves of the PBAT–PBS samples before, during and after degradation.

TGA curves and derivative thermogravimetric (DTG) curves of the PBAT–PLA blend samples show that thermal decomposition occurs in a two-step manner due to the incompatibility between the two polymer matrices (Figure 3a,c and Table 3). The onset thermal degradation temperatures for the PBAT–PLA samples exposed for 0, 30 and 60 days to industrial compositing conditions were 350 °C, 345 °C and 171 °C, respectively (as detailed in Table 3). The minor degradation peak of the PBAT–PLA blend occurred at 367.5 °C, corresponding to PLA, and the major degradation peak occurred at 415 °C, corresponding to PBAT. After 30 and 60 days of biodegradation, the minor degradation peak corresponding to PLA was reduced to 364 °C and 276 °C, respectively. Similarly, after 30 and 60 days of biodegradation, the major degradation peak corresponding to PBAT was reduced to 412 °C and 402 °C (Figure 3c and Table 3). These results demonstrate that, after 60 days of biodegradationthe blend PBAT-PLA significantly degraded, which indicates that the major PBAT component within the blend largely contributes to the biodegradation of the PBAT–PLA blend sample, and that the adopted industrial composting conditions can be used to facilitate the degradation of the PBAT–PLA blend.

The TGA curves and derivative (DTG) curves show that the thermal degradation onset temperatures of the PBAT–PBS samples exposed for 0, 30 and 60 days to industrial compositing conditions were 357 °C, 351 °C and 320 °C, respectively (Figure 3b,d and Table 3). The degradation of all samples occurred in a single major weight-loss step. This could be attributed to the strong compatibility between PBS and PBAT. After 30 and 60 days of biodegradation, the maximum degradation peaks of the PBAT–PBS blend decreased to 409.6 °C and 406.1 °C, respectively. It was also noticed that, after 60 days of biodegradation, the thermal degradation residues for both the PBAT–PLA and PBAT–PBS blends at 600 °C increased from 0.1% and 0.2% to 13.1% and 18.3%, respectively. The increase in residues could be due to the inorganic component of the soil in which the test samples were incubated. During the test, it was also noticed that the test samples were not visually distinguishable after 60 days of incubation in the compost. This could be indicative of the complete disintegration of the samples. This correlated well with the CO_2_ mineralization results demonstrating that the blends were highly degraded after 60 days of incubation.

### 3.4. The DSC Analyses

**Figure 4:**
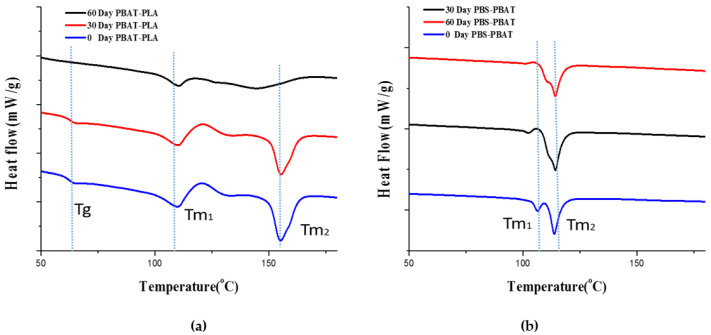
(**a**) The second heating scans of the PBAT–PLA samples before, during and after degradation, (**b**) and of the PBAT–PBS samples before, during and after degradation.

Figure 4 shows the DSC heating curves of the PBAT–PLA and PBAT–PBS blends before and after biodegradation. The corresponding glass transition temperatures (T_g_), cold crystallization temperatures (T_c_), % crystallinity (X_c_) and melting temperatures (T_m_) are summarized in Table 4.

Before biodegradation, the PBAT–PLA and PBAT–PBS blends displayed two melting peaks, corresponding to the individual polymers used to prepare the blends. This could be attributed to the blends being physical in nature rather than based on chemical interaction. A significant increase in the T_c_ values for the PBAT-PLA blend sample was observed after 60 days of biodegradation, when compared to before incubation and after 30 days of incubation. This increase could be explained by the faster degradation of the PBAT component of the blend in comparison to the highly crystalline PLA component. As the PBAT preferentially degrades, the percentage of PLA in the blend actually increases over the degradation period, and the cold crystallization temperature of the blend correspondingly increases as it approaches that of pure PLA. This preferential degradation of PBAT over PLA can also explain the increase in the degree of crystallinity of the PBAT-PLA blend in the degradation period between 30 days and 60 days.

A steady decrease in the degree of crystallinity for the PBAT–PBS blend indicated that the blend was gradually degrading over time. A steady decrease in T_c_ was also observed during the first 60 days. This was taken as an indication of the increasing amount of smaller crystal structures present within the sample as a result of degradation, hindering crystallization and thus lowering T_g_.

### 3.5. The XRD Analyses

**Figure 5:**
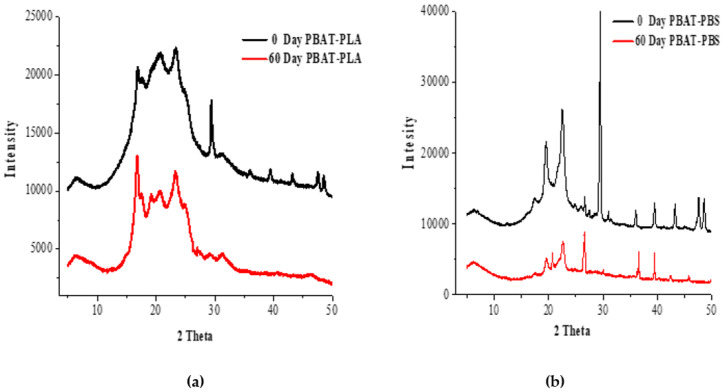
(**a**) XRD curves of the PBAT–PLA blend before and after degradation, and (**b**) XRD curves of the PBAT–PBS blend before and after degradation.

Figure 5 shows the XRD patterns for PBAT–PLA and PBAT–PBS before and after biodegradation.

Prior to degradation (Figure 5a), the PBAT–PLA blend displayed distinctive PBAT peaks at 2θ = 16.2°, 17.3°, 20.4° and 24.8°, and the peak at 2θ = 29.9° associated with the β-form crystals of PBAT [5]. PLA, being semi-crystalline in nature, displayed one sharp diffraction peak at ~24.8°, corresponding to the defective α-form crystal of PLA. The broad peak between 10° and 28° also belongs to the PLA polymeric matrix. These results indicated that PBAT was dispersed within the PLA. Pan et al. [34] studied the effect of MDI on the structure and mechanical properties of poly (lactic acid) and poly(butylene adipate-co-butylene terephthalate) blends, and deduced that the sample with 50 wt% of PBAT had reflections of PBAT crystals at 2θ = 16.2, 17.3, 20.4, 23.2 and 24.8° [34]. Post degradation, the XRD peaks of PBAT–PLA appeared to have shifted to lower 2θ values, which could be attributed to the reduction in the degree of crystallinity of the sample [5]. The PBAT–PBS blend, prior to compost incubation, had peaks similar to the PBAT–PLA sample, due to both blends mainly consisting of PBAT. The addition of PBS increased the intensity of the peaks at 2θ = 36.5°, 40.0°, 44.5°, 47.1° and 49.5°. It could be deduced that the presence of PBS improved the overall crystallinity. After biodegradation, the intensities of the peaks decreased, pointing to the microbial assimilation of low-molecular compounds mainly in the amorphous regions of the sample [5]. These results are consistent with the DSC results that showed that the crystallinity decreased as the number of biodegradation incubation days increased.

### 3.6. SEM Analyses

**Figure 6:**
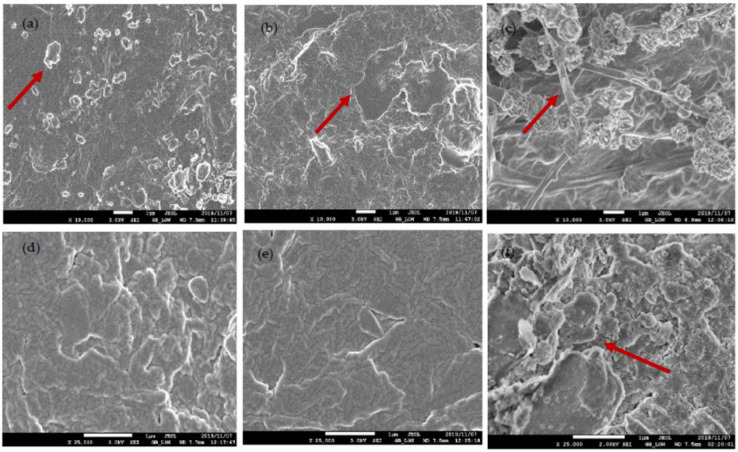
(**a**) SEM image of PBAT–PLA at day 0; (**b**) SEM image of PBAT–PLA after 30 days biodegradation; (**c**) SEM image of PBAT–PLA after 60 days of biodegradation; (**d**) SEM image of PBAT–PBS at day 0; (**e**) SEM image of PBAT–PBS after 30 days biodegradation; and (**f**) SEM image of PBAT-––S after 60 days of biodegradation.

Figure 6 shows the SEM images of the PBAT–PBS and PBAT–PLA blends before and after biodegradation. In Figure 6a, the PBAT–PLA blend morphology presented a ductile surface with granule-like spots, granting the material a rough exterior character. After the 30th day of biodegradation, the granules seemed to have disappeared, while small cracks appeared on the surface of the blend. The morphological changes as shown in arrow symbol, due to a biodegradation process, could clearly be observed after 60 days of exposure to (Figure 6c). This is evidenced by the formation of fibers on the surface of the blend. Weng et al. [35], in their study on the biodegradation behavior of PBAT, PLA and their blend under soil conditions, found that, after four months of degradation, the surface of the sample became more coarse, and the protruding rib was shallower than it was before degradation. From Figure 6d, it can be observed that, before the degradation process, the surface of the PBAT–PBS sample was relatively smooth with small fractures. After 30 days of exposure time, surface erosion as shown in arrow symbol was evidenced by the loss of the small polymer clusters on the film surface and the appearance of cracks in some regions of the PBAT–PBS sample. A more prominent microbial attack on the film can clearly be observed in Figure 6f, with more crevices evident on an uneven surface, indicating the degradation of the blend into smaller molecules.

### 3.7. Biodegradation under Home Composting Conditions

**Figure 7:**
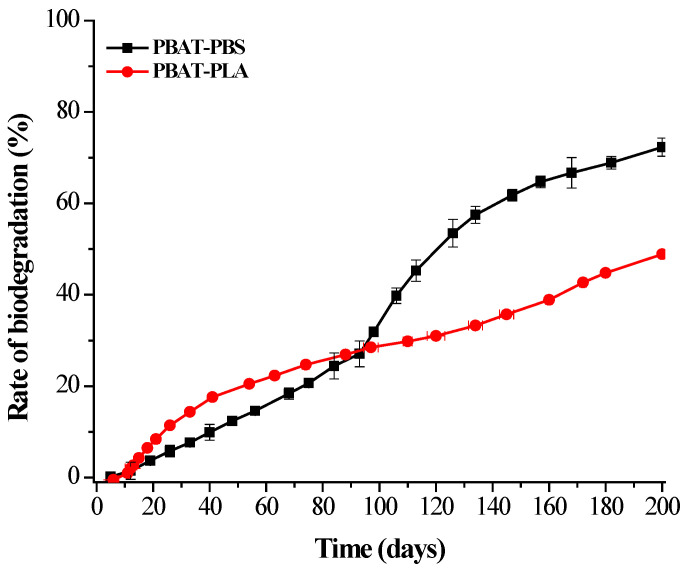
Biodegradation of the PBAT–PLA and PBAT–PBS blends under home composting conditions.

Figure 7 shows the rate of biodegradation of the PBAT-PBS and PBAT–PLA blends exposed to simulated home composting conditions. In this test, the temperature was kept at 28 °C. It was observed that both the PBAT–PLA and PBAT–PBS blends underwent a slower biodegradation rate, reaching 28% and 50%, respectively, after the first 120 days. Thereafter, a slight exponential phase was observed for the PBAT–PBS blend, with a degree of biodegradation of about 72% reached after the 200 days. The degree of biodegradation for PBAT–PLA reached a plateau at 50% after 200 days. This indicated that about 6–7 months were required to reach 70% biodegradation of the PBAT–PBS sample and 50% of the PBAT–PLA sample under home composting conditions, when compared to industrial composting conditions.

It has been reported that under industrial composting conditions, the rates of biodegradation are higher due to incubation conditions, such as a higher moisture content (>50%), aeration (>6%) and temperatures of 58–60 °C. These conditions allow for the hydrolytic degradation of materials due to the activity of thermophilic microbes, when compared to mesophilic microbes that are responsible for hydrolytic degradation under home composting conditions [36]. The activity of thermophilic microbes is increased due to the higher ambient temperatures [36,37], thus leading to increased growth rates of microbial populations. The larger microbial populations are then able to assimilate polymeric carbon at an increased rate, when compared to assimilation by relatively smaller populations cultivated under home composting conditions. It also has been reported that, when operating the composting process at 58–60 °C, PLA undergoes hydrolytic degradation into low molecular weight compounds that are easily assimilated by microorganisms present in the compost medium [24]. This can be ascribed to the fact that the PLA glass transition temperature falls within these temperatures, thus the movement of polymer chain molecules is enhanced, and hydrolytic cleavage can occur. The studies showed that no significant degradation was observed for PLA under natural soil and home composting conditions for an incubation period of 12 months [5,24]. The present study reveals that the PBAT–PLA blend under home composting conditions achieves 50% biodegradation within 200 days of incubation. The obtained results are mainly attributed to the biopolymer blending process, with the PBAT/PLA(70/30) blend assisting in altering the crystalline nature of the 30% PLA content in the PBAT matrix. This process contributes to transforming more amorphous groups in the PBAT/PLA blend, which can then easily undergo biodegradation under home composting conditions (Figure 7). In addition, the physical–chemical properties of the compost after the biodegradation test (compost + test sample) were analyzed in comparison to the original compost (blank), but no significant changes were observed on the pH, total dry solids and volatile contents (Table 1). The obtained results indicate that the polymer degradation end products are not affected by the physical–chemical properties of the compost. Despite this, a further investigation of the eco-toxicology and environmental safety are required for composted materials to define the fact that compost does not contain any environmentally harmful substances.

## 4. Conclusions

The results show that both the PBAT–PLA and PBAT–PBS blends experienced higher rates of biodegradation when exposed to an industrial composting environment, when compared to a home composting environment. The analyses of the amounts of evolved CO_2_ from both blends indicate that the PBAT–PLA blend experienced a longer lag phase, in comparison to that of the PBAT–PBS blend. This was mainly attributed to the biodegradation occurring via a two-step mechanism, with the initial hydrolysis of PLA chains followed by a microbial attack. The FT-IR results confirm that both the PBAT–PLA and PBAT–PBS blends show changes in the carbonyl and hydroxyl groups under composting conditions, which is largely a result of hydrolysis biodegradation. The TGA results indicate that the PBAT–PLA blend underwent a degradation process, which was mainly ascribed to the incompatibility of PLA with PBAT. Additionally, the results show a significant weight reduction in the PBAT–PLA blend after only 60 days of biodegradation. The TGA results of the PBAT–PBS blend show a single-step degradation peak and significant weight reduction observed after 30 days of biodegradation. The DSC analyses showed a decrease in the degree of crystallinity of the PBAT–PLA blend after biodegradation, and this phenomenon was complemented by the disappearance of the XRD peaks of the blend. Similarly, the intensities of the XRD peaks of the PBAT–PBS blend significantly decreased after biodegradation, clearly demonstrating that microbial assimilation had taken place. The changes in surface morphology, due to microbial attacks, for both blends were captured by SEM imaging, further proving that a significant degradation of the blends had taken place. In summary, the obtained results clearly indicate that the physical and thermal characteristics of the PBAT–PLA and PBAT–PBS blends, such as the functional groups present, greatly influence the mechanisms by which degradation occurs. The biodegradation of both blends was more extensively enhanced under industrial composting conditions, when compared to home composting conditions.

## Figures and Tables

**Table 1:** Properties of the controlled compost.

Analysis	Compost
Total dry solids (%) ^1^	55
Volatile solids (%) ^2^	53
pH of the compost solution	7.1
Total organic carbon content (%)	10.6
Total nitrogen (%)	0.9
Carbon/nitrogen ratio	11.8

^1^ The amount of total dry solids obtained by drying a known volume of compost at approximately 105 °C for 10 h. ^2^. The amount of volatile solids obtained by subtracting the residue of a known volume of compost after incineration at approximately 550 °C.

**Table 2:** Carbonyl index (I_co_) for the PBAT–PLA and PBAT–PBS blends.

Number of Days	C–O (Wavenumber at 1710–1712 cm^−1^)	C–H (Wavenumber at 1452 cm^−1^)	I_co_
PBAT–PLA			
0	64.89	11.86	4.41
30	66.14	12.06	5.48
60	222.96	12.56	9.88
PBAT–PBS			
0	50.42	10.18	4.95
30	45.12	10.35	4.36
60	34.92	10.33	3.32

**Table 3:** TGA results for the PBAT–PLA and PBAT–PBS blends, as well as pure PLA, PBAT and PBS.

Sample	Degradation Period (Days)	T_onset_ (°C)	T_minor_ (°C)	T_max_ (°C)	Residue (%)
PLA	0	321.6		369.4	0.1
PBAT	0	356.4	-	406.4	0.1
PBS	0	358.0	-	399.6	0.1
PBAT–PLA	0	350.0	-	415.3	0.6
	30	346.9	367.5	411.7	0.4
	60	171.0	363.5	402.1	13.1
PBAT–PBS	0	356.5	276.2	409.2	0.2
	30	351.1	-	409.6	2.0
	60	320.3	-	406.1	18.3

**Table 4:** DSC results for the PBAT–PLA and PBAT–PBS blends, as well as pure PLA, PBAT and PBS.

Sample	Degradation Period (Days)	T_g_ (°C)	T_c_ (°C)	∆H (J/g)	T_m_ (°C)	X_c_ (%)
PLA	0	59.8	118.5	31.8	152.6; 160.3	33.94
PBAT	0	n.d	69.8	14.8	121.4	12.98
PBS	0	n.d	82.8	66.0	116.3	59.79
PBAT–PLA	0	59.64	51.98	15.8	110.4; 155.1	13.86
	30	59.1	56.2	10.5	110.6; 155.3	9.25
	60	n.d	78.2	14.4	109.9; 143.8	12.63
PBAT–PBS	0	n.d	90.3	45.9	105.9; 114.0	41.62
	30	n.d	85.4	42.9	114.2	38.90
	60	n.d	78	28.5	112.4	25.85

## Data Availability

The datasets generated during and/or analyzed during the current study are available from the corresponding author on reasonable request.
